# Multi-lead pacing for cardiac resynchronization therapy in heart failure: a meta-analysis of randomized controlled trials

**DOI:** 10.1093/ehjopen/oeac013

**Published:** 2022-02-26

**Authors:** Mark K Elliott, Vishal Mehta, Nadeev Wijesuriya, Baldeep S Sidhu, Justin Gould, Steven Niederer, Christopher A Rinaldi

**Affiliations:** School of Biomedical Engineering and Imaging Sciences, King's College London, St Thomas' Hospital, Westminster Bridge Road, London, SE1 7EH, UK; Department of Cardiology, Guy's and St Thomas' NHS Foundation Trust, Westminster Bridge Road, London, SE1 7EH, UK; School of Biomedical Engineering and Imaging Sciences, King's College London, St Thomas' Hospital, Westminster Bridge Road, London, SE1 7EH, UK; Department of Cardiology, Guy's and St Thomas' NHS Foundation Trust, Westminster Bridge Road, London, SE1 7EH, UK; School of Biomedical Engineering and Imaging Sciences, King's College London, St Thomas' Hospital, Westminster Bridge Road, London, SE1 7EH, UK; Department of Cardiology, Guy's and St Thomas' NHS Foundation Trust, Westminster Bridge Road, London, SE1 7EH, UK; School of Biomedical Engineering and Imaging Sciences, King's College London, St Thomas' Hospital, Westminster Bridge Road, London, SE1 7EH, UK; Department of Cardiology, Guy's and St Thomas' NHS Foundation Trust, Westminster Bridge Road, London, SE1 7EH, UK; School of Biomedical Engineering and Imaging Sciences, King's College London, St Thomas' Hospital, Westminster Bridge Road, London, SE1 7EH, UK; Department of Cardiology, Guy's and St Thomas' NHS Foundation Trust, Westminster Bridge Road, London, SE1 7EH, UK; School of Biomedical Engineering and Imaging Sciences, King's College London, St Thomas' Hospital, Westminster Bridge Road, London, SE1 7EH, UK; School of Biomedical Engineering and Imaging Sciences, King's College London, St Thomas' Hospital, Westminster Bridge Road, London, SE1 7EH, UK; Department of Cardiology, Guy's and St Thomas' NHS Foundation Trust, Westminster Bridge Road, London, SE1 7EH, UK

**Keywords:** Multi-lead pacing, Triventricular pacing, Multi-site pacing, Cardiac resynchronization therapy, Heart failure

## Abstract

**Aims:**

Multi-lead pacing is a potential therapy to improve response to cardiac resynchronization therapy (CRT) by providing rapid activation of the myocardium from multiple sites. Here, we perform a meta-analysis of randomized controlled trials to assess the efficacy of multi-lead pacing.

**Methods and results:**

A literature search was performed which identified 251 unique records. After screening, 6 studies were found to meet inclusion criteria, with 415 patients included in the meta-analysis. Four studies performed multi-lead pacing with two left ventricular (LV) leads and one right ventricular (RV) lead. One study used two RV leads and one LV lead, and one study used both configurations. There was no difference between multi-lead pacing and conventional CRT in LV end-systolic volume [mean difference (MD) −0.54 mL, *P* = 0.93] or LV ejection fraction (MD 1.42%, *P* = 0.40). There was a borderline significant improvement in Minnesota Living With Heart Failure Questionnaire score for multi-lead pacing vs. conventional CRT (MD −4.46, *P* = 0.05), but the difference was not significant when only patients receiving LV-only multi-lead pacing were included (MD −3.59, *P* = 0.25). There was also no difference between groups for 6-min walk test (MD 15.06 m, *P* = 0.38) or New York Heart Association class at follow-up [odds ratio (OR) 1.49, *P* = 0.24]. There was no difference in mortality between groups (OR 1.11, *P* = 0.77).

**Conclusion:**

This meta-analysis does not support the use of multi-lead pacing for CRT delivery. However, significant variation between studies was noted, and therefore a benefit for multi-lead pacing in select patients cannot be excluded, and further investigation may be warranted.

## Graphical Abstract

**Graphical Abstract:**
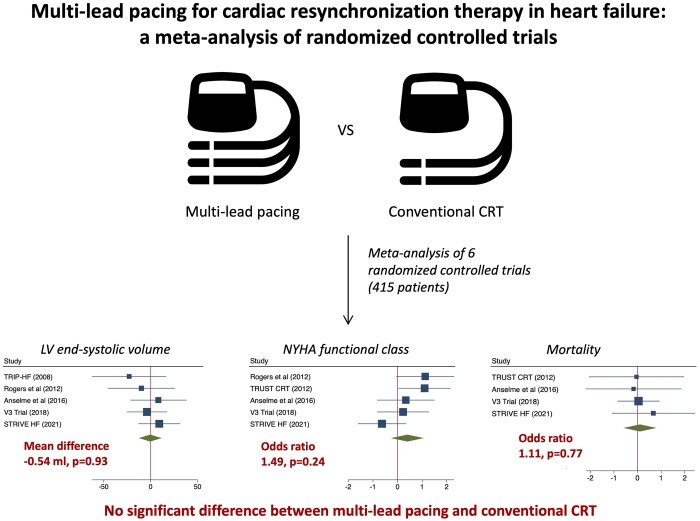

## Introduction

Cardiac resynchronization therapy (CRT) is a well-established, effective therapy for patients with dyssynchronous heart failure, however, 30% of patients fail to improve after implantation.^1^ Non-response to CRT is likely to be multi-factorial, comprising poor patient selection, sub-optimal left ventricular (LV) lead placement, ineffective CRT delivery, and sub-optimal optimization of device programming. Multi-site pacing has been proposed as a potential technique to allow stimulation of a larger volume of myocardium, thus achieving more rapid electrical activation and resynchronization.^2^ It may also increase the chance of stimulating the latest site of activation which may provide benefit in ischaemic cardiomyopathy by ensuring regions of scar are bypassed to allow more effective activation of viable myocardium. This can be achieved with multi-point pacing using a quadripolar lead, where LV pacing is performed via stimulation from multiple electrodes within the same lead. While early studies of multi-point pacing demonstrated promising improvements in short-term haemodynamic and dyssynchrony outcomes,^3–5^ a recent meta-analysis of multi-point pacing found no significant benefit over conventional CRT when only randomized studies were included.^6^

Multi-lead pacing is an alternative to multi-point pacing, and theoretically allows the recruitment of a larger volume of myocardium, as greater separation between pacing electrodes can be achieved.^7^ Multi-lead pacing has been performed with different combinations of right ventricular (RV) and LV leads: either two RV leads and one LV lead, or one RV lead and two LV leads. Early mechanistic and feasibility studies showed potential benefits of both strategies of multi-lead pacing,^8–14^ and subsequent small randomized controlled trials (RCTs) demonstrated superior symptomatic and echocardiographic response with multi-lead CRT compared to conventional biventricular pacing.^15–18^ A previous meta-analysis, which included both randomized and non-randomized studies, demonstrated a greater improvement in LV ejection fraction and New York Heart Association (NYHA) class with multi-lead pacing compared to conventional CRT.^19^ Two large multi-centre RCTs have since been performed: the V^3^-trial^20^ and STRIVE-HF,^21^ which both showed no benefit for multi-lead pacing. In this study, we performed a meta-analysis of RCTs to determine if multi-lead pacing provides a benefit over conventional biventricular CRT.

## Methods

### Literature search and selection criteria

The systematic review and meta-analysis was performed in accordance with the PRISMA statement.^22^ A literature search was performed on Medline, EMBASE and Cochrane CENTRAL databases up to October 2021 using keywords ‘multi site pacing’, ‘triventricular pacing’, and ‘triple site pacing’. In addition, references of previously published meta-analyses, review articles, pre-prints, letters, and editorials were searched. Two authors (M.K.E. and V.M.) performed both the initial title/abstract screen, and full text review independently. The major inclusion criteria were RCTs comparing the efficacy of multi-lead pacing vs. standard biventricular CRT and follow-up period ≥3 months. Observational studies, non-randomized studies, case reports, review articles, and studies with only acute haemodynamic outcome data were excluded.

### Data extraction

Data from included studies were extracted by two reviewers independently (M.K.E. and N.W.). Data recorded included trial design, number of patients, inclusion and exclusion criteria, baseline patient characteristics, and outcomes of pre-specified efficacy endpoints. The potential for bias for each eligible study was assessed using version 2 of the Cochrane Risk of Bias Tool (RoB) by two reviewers independently (M.K.E. and N.W.). Risk of bias was assessed separately for echocardiographic and symptomatic endpoints.^23^

### Meta-analysis

Statistical analyses and creation of forest plots was performed using the Stata 16 software package (StataCorp. 2019. *Stata Statistical Software: Release 16*. College Station, TX, USA: StataCorp LLC) using the ‘*meta*’ command. Mean difference (MD) with 95% confidence intervals (CIs) were computed for continuous variables. Outcome data expressed as improvement from baseline was used preferentially if available but studies that reported absolute values at follow-up only (including cross-over studies) were still included and outcome data were combined using unstandardized MD with 95% CIs.^24^ The odds ratio (OR) with 95% CI were computed for categorical variables. Significant heterogeneity was anticipated, and so a random effects meta-analysis model was used for all analyses. Intention-to-treat data were used wherever possible. Sub-group analysis was performed for the configuration of two LV leads and one RV lead. Heterogeneity was assessed by the Cochran Q test, where significant heterogeneity was defined as *P* < 0.1, and *I*^2^ where heterogeneity was considered low, moderate, and high for *I*^2^ values of <30%, 30–60% and >60%, respectively.^25^ A *P*-value <0.05 for the MD or OR of efficacy endpoints was considered significant.

## Results

**Figure 1:**
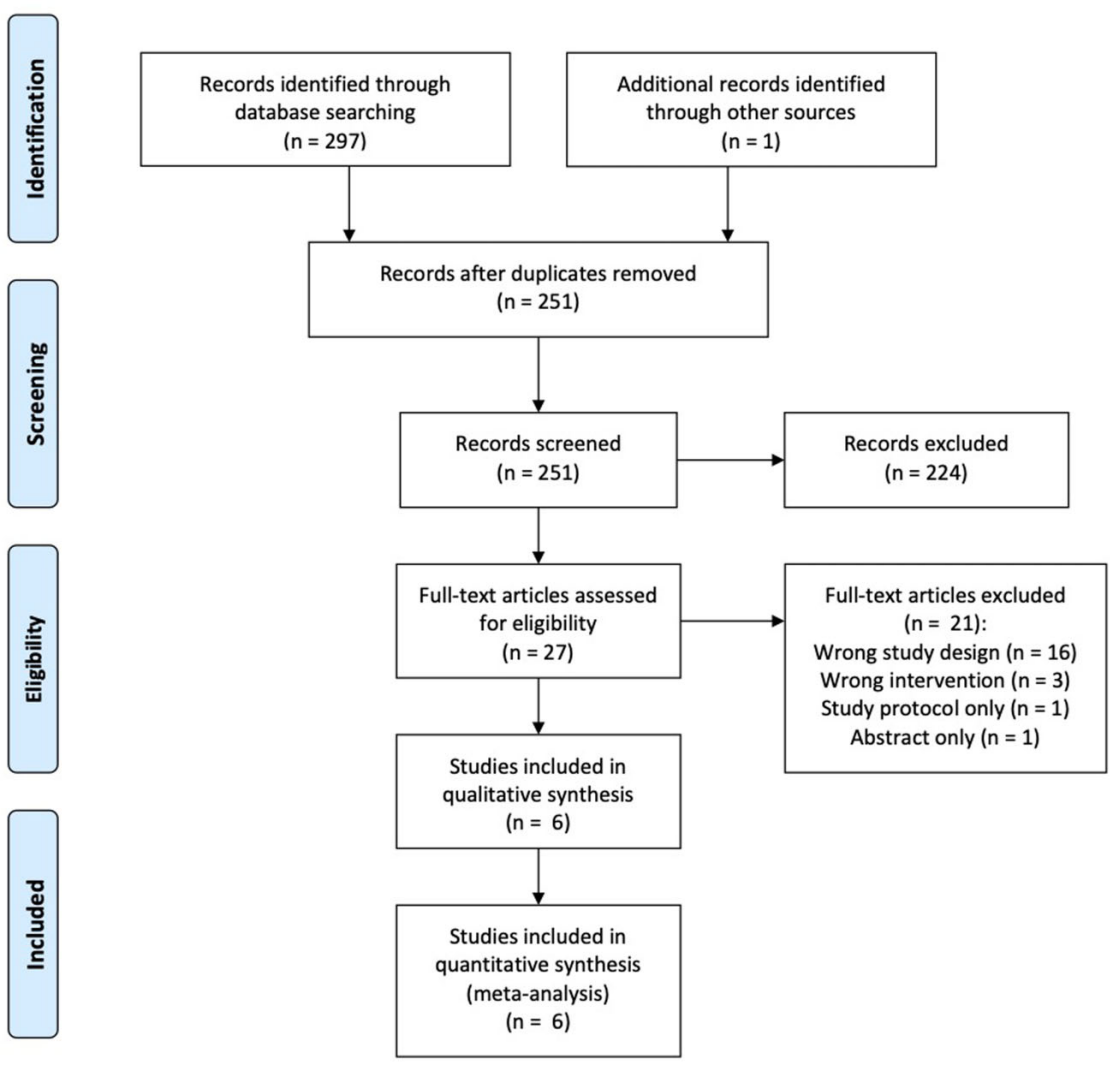
CONSORT flow diagram.

**Figure 2:**
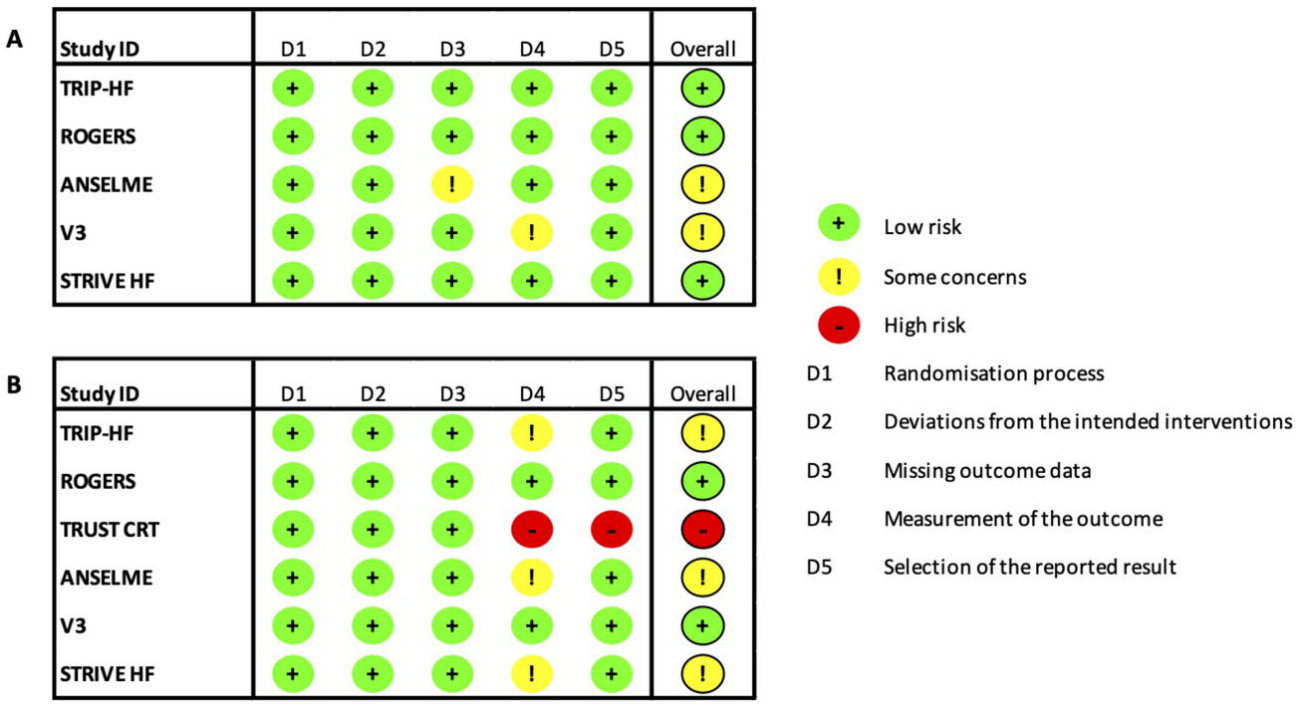
Cochrane Risk of Bias 2 assessment for echocardiographic (*A*) and symptomatic (*B*) endpoints.

251 unique records were identified in the initial search, of which 224 were excluded after initial title/abstract screen. Twenty-seven full text articles were assessed for eligibility and six were found to meet inclusion criteria and were included in the quantitative synthesis (*Figure 1*). A total of 415 patients were included in the meta-analysis. Two trials were cross-over RCTs,^15^^,^^17^ and four were parallel RCTs.^16^^,^^18^^,^^20^^,^^21^ Four of the studies were multi-centre. Inclusion criteria varied between studies and are summarized in *Table 1*. Four studies assessed multi-lead pacing with two LV leads and one RV lead^17^^,^^18^^,^^20^^,^^21^ and one trial assessed multi-lead pacing with one LV lead and two RV leads.^16^ The study by Rogers *et al*.^15^ included both combinations in different groups: two LV leads and one RV lead (Group A) and one LV lead and two RV leads (Group B). The study by Anselme *et al*.^16^ reported outcome data for all enrolled patients at 6 months (*n* = 76), which was included in the meta-analysis. The study also reported further outcome data for a smaller subset of patients at 12 months (*n* = 40), which was not included. The full results of the TRUST-CRT trial as described in the protocol have not been published^26^; however, a sub-study which reported on implantation feasibility, adverse events and lead performance has been published and contained limited efficacy outcome data (mortality and NYHA class) and these were included in the meta-analysis.^18^ Baseline patient characteristics are demonstrated in *Table 2*. The risk of bias assessment is demonstrated graphically in *Figure 2* and was performed separately for echocardiographic and symptomatic endpoints.

**Table 1:** Characteristics of trials included in the meta-analysis

Study	Single or multi-centre	Design	Subjects (*n*)	Inclusion criteria	Pacing leads
TRIP-HF (2008)	Multi-centre	Crossover RCT	26	NYHA class III–IV Permanent AF requiring cardiac pacing LVEF ≤35%	2 LV leads and 1 RV lead
Rogers *et al*. (2012)	Single centre	Crossover RCT	37	NYHA class II–IV LVEF ≤35% QRS duration ≥150 ms (or <150 ms with echo evidence of dyssynchrony)	Two groups: A: 2 LV leads and 1 RV lead B: 1 LV lead and 2 RV leads
TRUST CRT Substudy (2012)	Single centre	Parallel RCT	98	NYHA class III–IV LVEF ≤35% and significant mechanical dyssynchrony on echo QRS duration >120 ms Sinus rhythm	2 LV leads and 1 RV lead
Anselme *et al*. (2016)	Multi-centre	Parallel RCT	76	NYHA class II–IV LVEF ≤35% QRS duration >120 ms for NYHA class III-IV and >150 ms for NYHA class II Sinus rhythm	1 LV lead 2 two RV leads
V^3^ trial (2018)	Multi-centre	Parallel RCT	83	Non-responders after 6 months of CRT (defined as unchanged or worsened CCS) NYHA II–III, LVEF ≤35% and QRS >120 ms at time of CRT implant	2 LV leads and 1 RV lead
STRIVE-HF (2021)	Multi-centre	Parallel RCT	95	NYHA class II–IV LVEF ≤35% LBBB and QRS 120-150 ms	2 LV leads and 1 RV lead

AF, atrial fibrillation; CCS, clinical composite score; CRT, cardiac resynchronization therapy; HF, heart failure; LBBB, left bundle branch block; LV, left ventricular; LVEF, left ventricular ejection fraction; NYHA, New York Heart Association; RCT, randomized controlled trial; RV, right ventricular.

**Table 2:** Baseline characteristics of patients included in the meta-analysis

Study	Age (years)	Male (%)	ICM (%)	AF (%)	QRSd (ms)	LVEF (%)	LVEDV (mL)	LVESV (mL)	ACEi/ARB (%)	Beta-blocker (%)	MRA (%)
TRIP-HF (2008)	70 ± 8	100	27	100	159 ± 47	24 ± 11	197 ± 68	154 ± 68	96	73	NR
Rogers *et al*. (2012)	66.4 ± 11.4	81.4	62.8	14.0	138.8 ± 32.6	23.4 ± 6.7	253 ± 87.5	196.3 ± 80.4	98	81	51
TRUST CRT Substudy (2012)	61.8 ± 9.2	78.6	61.2	14.2^a^	167.0 ± 24.3	23.7 ± 4.4	273.7 ± 97.1	209.3 ± 79.4	99	99	96
Anselme *et al*. (2016)	69.4 ± 10.7	71.1	43.4	26.3^a^	162 ± 21	28.7 ± 6.3	202 ± 70.7	145 ± 58.9	NR	NR	NR
V^3^ trial (2018)	71.3 ± 7.8	86.7	55.4	60.2	160 ± 36.8 (paced)	26.4 ± 6.4	213 ± 75.7	151 ± 59.8	88	90	41
STRIVE-HF (2021)	68.4 ± 9.8	75.8	57.9	24.2	136.5 ± 8.6	26.7 ± 6.8	189.5 ± 76.2	142.8 ± 67.6	94.5	93.6	79.7

ACEi, angiotensin converting enzyme inhibitor; AF, atrial fibrillation; ARB, angiotensin receptor blocker; ICM, ischaemic cardiomyopathy; LVEDV, left ventricular end-diastolic volume; LVEF, left ventricular ejection fraction; LVESV, left ventricular end-systolic volume; MRA, mineralocorticoid receptor antagonist; NR, not reported; QRSd, QRS duration.

a Paroxysmal atrial fibrillation only.

### Echocardiographic endpoints

**Figure 3:**
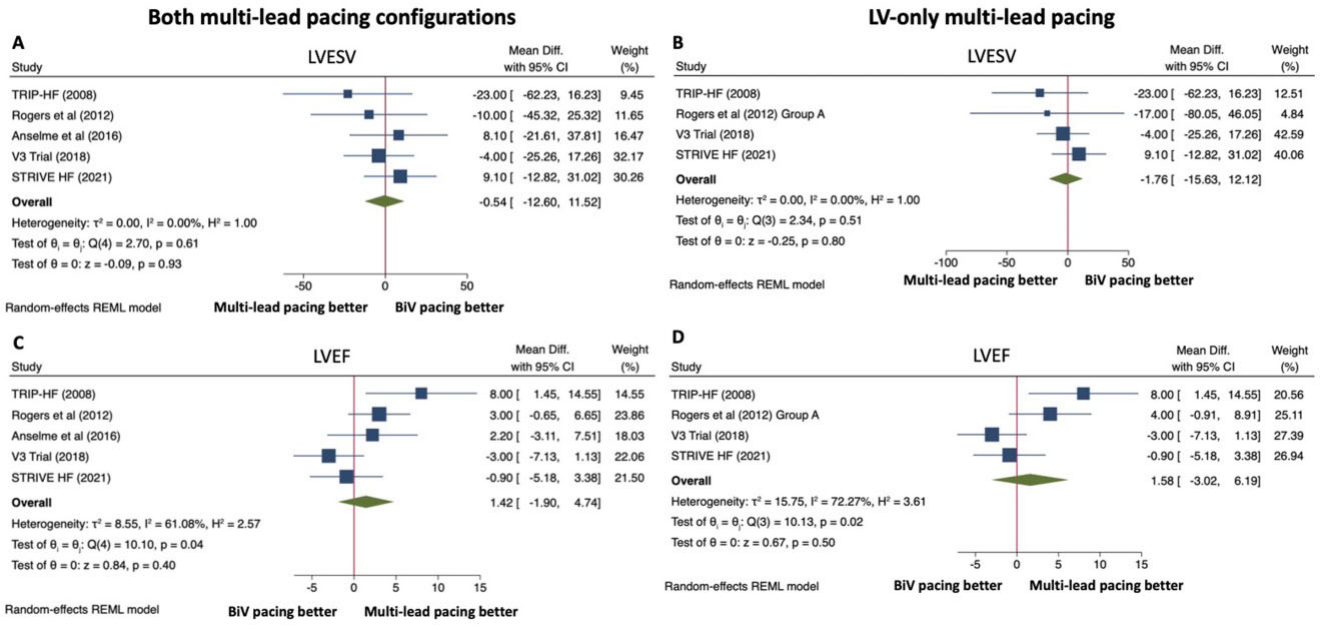
Forest plots for echocardiographic endpoints. Effect of multi-lead pacing vs. conventional biventricular (BiV) pacing on left ventricular end-systolic volume (LVESV) for both multi-lead pacing configurations (*A*) and for patients with left ventricle (LV)-only multi-lead pacing (*B*). Effect of multi-lead pacing vs. biventricular pacing on left ventricular ejection fraction (LVEF) for patients with both multi-lead pacing configurations (*C*) and for patients with LV-only multi-lead pacing (*D*). CI, confidence intervals; mean diff, mean difference; REML, restricted maximum likelihood.

Five studies reported echocardiographic outcomes (*Figure 3*). There was no difference between multi-lead pacing and conventional biventricular CRT in LV end-systolic volume (LVESV) at follow-up when both multi-lead pacing configurations were included [MD −0.54 mL, 95% CI (−12.60, 11.52), *P* = 0.93]. Heterogeneity was low between studies (*P* = 0.61, *I*^2^ = 0.00%). For LV-only multi-lead pacing (two LV leads and one RV lead), there was also no difference in LVESV [MD −1.76 mL, 95% CI (−15.63, 12.12), *P* = 0.80] with low heterogeneity between studies (*P* = 0.51, *I*^2^ = 0.00%). Similarly, there was no difference in LV ejection fraction between groups for both pacing configurations [MD 1.42%, 95% CI (−1.9, 4.74), *P* = 0.40] and for LV-only multi-lead pacing [MD 1.58%, 95% CI (−3.02, 6.19), *P* = 0.50]; however, heterogeneity between studies was high (*P* = 0.04, *I*^2^ = 61.08 and *P* = 0.02, *I*^2^ = 72.27%, respectively).

### Symptomatic endpoints

**Figure 4:**
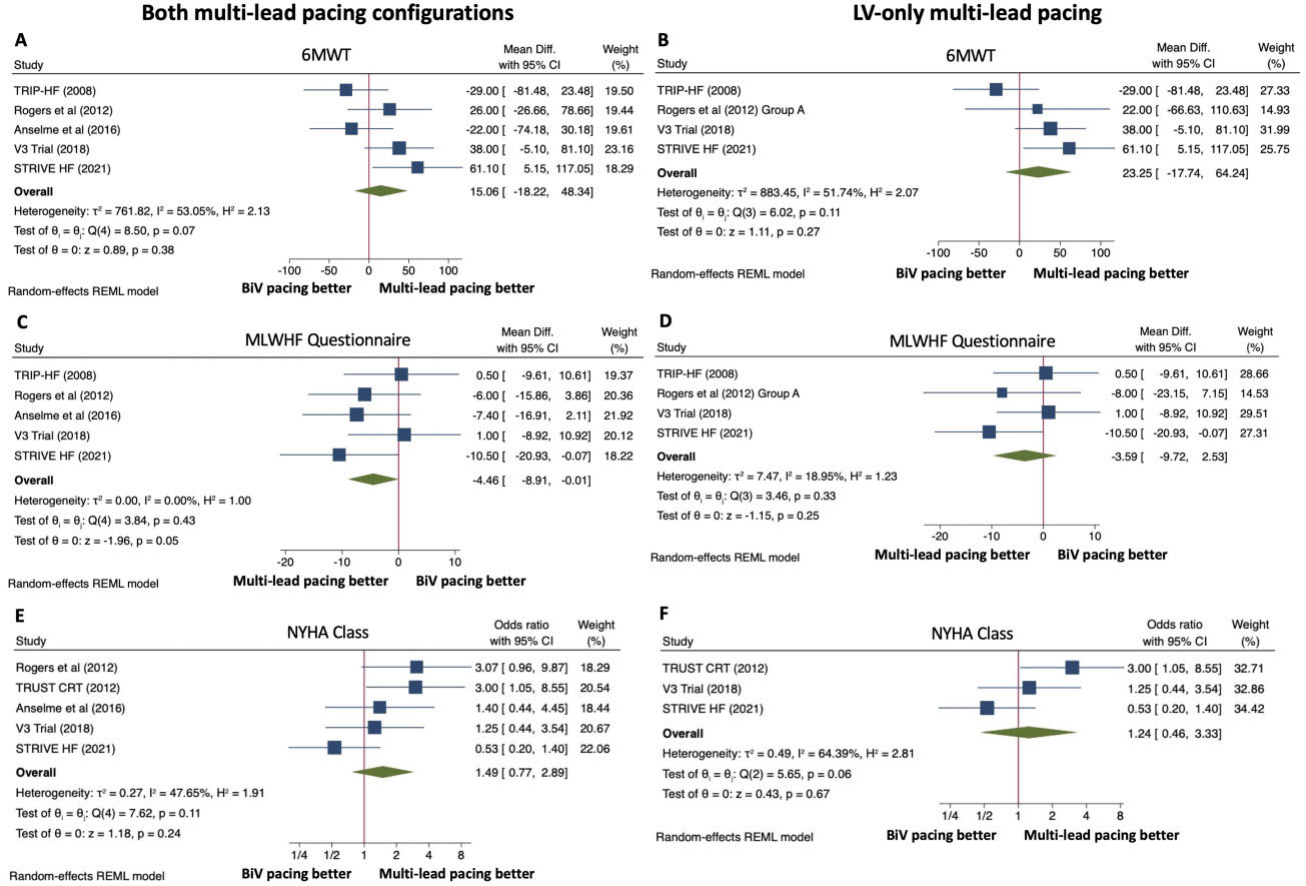
Forest plots for symptomatic endpoints. Effect of multi-lead pacing vs. conventional biventricular (BiV) pacing on 6-minute walk test (6MWT) for both multi-lead pacing configurations (*A*) and for patients with left ventricle (LV)-only multi-lead pacing (*B*). Effect of multi-lead pacing vs. BiV pacing on Minnesota Living With Heart Failure (MLWHF) questionnaire score for patients with both multi-lead pacing configurations (*C*) and for patients with LV-only multi-lead pacing (*D*). Effect of multi-lead pacing vs. BiV pacing on the proportion of patients in New York Heart Association (NYHA) Class 3 or 4 at follow-up for patients with both multi-lead pacing configurations (*E*) and for patients with LV-only multi-lead pacing (*F*). CI, confidence intervals; mean diff, mean difference; REML, restricted maximum likelihood.

Five studies reported symptomatic outcomes using 6-min walk test (6MWT), the Minnesota Living with Heart Failure Questionnaire (MLWHF) and NYHA class (*Figure 4*). There was no difference in 6MWT performance between multi-lead pacing and conventional CRT for both lead configurations [MD 15.06 m, 95% CI (−18.22, 48.34), *P* = 0.38] and for LV-only multi-lead pacing [MD 23.25 m, 95% CI (−17.74, 64.24), *P* = 0.27], with a moderate degree of heterogeneity between studies (*P* = 0.07, *I*^2^ = 53.05% and *P* = 0.11, *I*^2^ = 51.74%, respectively). When both multi-lead pacing configurations were included, there was a lower symptom burden measured via MLWHF questionnaire for multi-lead pacing compared to conventional CRT and this reached borderline statistical significance [MD −4.46, 95% CI (−8.91, −0.01), *P* = 0.05] with low heterogeneity between studies (*P* = 0.43, *I*^2^ = 0.00%). However, there was no significant difference for LV-only multi-lead pacing [MD −3.59, 95% CI (−9.72, 2.53), *P* = 0.25], with heterogeneity between studies also low (*P* = 0.33, *I*^2^ = 18.95%). There was no difference between groups in the proportion of patients who were NYHA class III or IV at follow-up for both lead configurations [OR 1.49, 95% CI (0.77, 2.89), *P* = 0.24] and for LV-only multi-lead pacing [OR 1.24, 95% CI (0.46, 3.33), *P* = 0.67], though heterogeneity between studies was moderate (*P* = 0.11, *I*^2^ = 47.65%) and high (*P* = 0.06, *I*^2^ = 64.39%), respectively.

### Mortality

**Figure 5:**
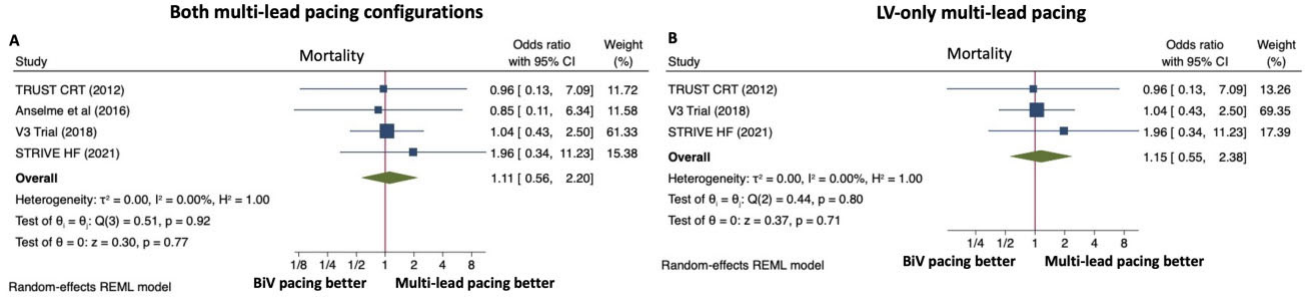
Forest plots for mortality endpoint. Effect of multi-lead pacing vs. conventional biventricular (BiV) pacing on mortality for both multi-lead pacing configurations (*A*) and for patients with left ventricle (LV)-only multi-lead pacing (*B*). CI, confidence intervals; REML, restricted maximum likelihood.

Mortality outcomes were reported for the four parallel RCTs (*Figure 5*). There was no difference in mortality between multi-lead pacing and conventional CRT for both lead configurations [OR 1.11, 95% CI (0.56, 2.20), *P* = 0.77] and for LV-only multi-lead pacing [OR 1.15, 95% CI (0.55, 2.38), *P* = 0.71]. Heterogeneity was low between studies for both analyses (*P* = 0.92, *I*^2^ = 0.00% and *P* = 0.80, *I*^2^ = 0.00%, respectively).

## Discussion

### Main findings

We report the largest meta-analysis of RCTs on the use of multi-lead pacing for CRT to date. We found no significant difference in LV ejection fraction or end-systolic volumes at follow-up between multi-lead pacing or conventional biventricular CRT. There was a borderline-significant improvement in patient-reported symptoms via the MLWHF questionnaire in the multi-lead pacing group compared to conventional CRT, however there was no difference when only patients who received LV-only multi-lead pacing were included. Moreover, only two of the studies (Rogers *et al*. and Anselme *et al*.)^15^^,^^16^ reported blinding of the patients to treatment allocation, and therefore significant bias in questionnaire scoring secondary to a placebo effect is possible. There was no difference between multi-lead pacing and conventional CRT when other assessments of symptoms (6MWT and NYHA class) were used. There was also no difference in mortality at follow-up between groups. These results differ from the previously published meta-analysis on multi-lead pacing.^19^ Unlike our analysis, the previous study included observational studies, which significantly increases the risk of bias, and was performed before the publication of the V^3^ trial^20^ and STRIVE-HF,^21^ which are the largest randomized trials of multi-lead pacing performed to date.

There are significant drawbacks to a multi-lead pacing approach. Placing an additional lead, particularly within the coronary sinus, can be technically challenging, and inevitably increases procedural and fluoroscopy times. Multi-site pacing is associated with higher rates of battery depletion,^27^^,^^28^ with associated costs and risks of more frequent generator replacement procedures. The use of a Y-connector is associated with raised pacing thresholds, which can further decrease battery longevity.^29^^,^^30^ While a dedicated triventricular CRT device with an internal Y-connector has been developed (Paradym TriV CRT-D, MicroPort CRM, Clarmart, France) and may overcome this issue, its use in the STRIVE-HF trial was still associated with significantly lower battery longevity compared to conventional biventricular CRT (5.5 ± 2.3 vs. 8.6 ± 2.7 years; *P* < 0.001).^21^ The drawbacks of multi-lead pacing must be overcome by significant benefits to supports its clinical use, and these were not demonstrated in our analysis.

There is evidence from mechanistic and animal model studies that the benefits of multi-lead pacing may be restricted to patients who do not achieve an optimal response from conventional CRT with a single LV lead. In an invasive haemodynamic study of 16 patients, only acute non-responders had an incremental benefit with multi-lead pacing over conventional CRT.^12^ This is supported by animal model studies of left bundle branch block (LBBB), in which acute haemodynamic improvements with multi-lead pacing were only seen in cases where the response to single-site LV pacing was suboptimal.^31^^,^^32^ This suggests that if an LV lead can be placed in the optimal position, the benefit of an additional LV lead may be minimal. In the studies that reported LV lead position, a high proportion of primary LV leads were placed in a lateral branch of the coronary sinus. In STRIVE-HF, 89.6% of patients in the conventional CRT group, and 97.8% of patients in the multi-lead pacing group had a primary LV lead in a lateral vein.^21^ Similarly, all patients in the study by Rogers *et al*.,^15^ and 97% of patients in the study by Leclercq *et al*.^17^ had primary LV leads placed in a lateral vein. The presence of significant lateral wall scar may also affect response to CRT, and in a previous mechanistic study of 24 patients, a benefit for multi-point pacing was only found in a small sub-group of patients with a significant burden of LV scar.^33^ None of the included studies reported the presence or location of myocardial scar. STRIVE-HF was the only study to perform a sub-analysis by aetiology of heart failure, however no benefit for multi-lead pacing was found in patients with ischaemic aetiology.^21^

### Variability of included studies

The studies included in this meta-analysis were very heterogeneous in terms of the patient cohort, study design and pacing lead configuration. The inclusion criteria varied markedly between studies. The TRIP-HF trial only included patients with permanent atrial fibrillation (AF) who required pacing and had severe LV systolic impairment.^16^ Patients with AF are known to have an attenuated benefit from CRT^34^ and cannot achieve atrioventricular resynchronization which is known to be an important component of the benefit received from CRT, particularly in non-LBBB patients.^35–37^ This cohort would also include patients without underlying electrical dyssynchrony during intrinsic rhythm which is a different cohort to patients with dyssynchronous heart failure who meet the conventional indications for CRT.^38^^,^^39^ Interestingly, this study demonstrated significant improvements in both symptomatic and echocardiographic endpoints for multi-lead pacing over conventional CRT. The studies by Anselme *et al*.^16^ and Rogers *et al*.^15^ and the TRUST CRT study^18^ included patients with more conventional CRT indications, though the latter two studies included echo dyssynchrony metrics as a requirement for at least some of the patients, and the former study mandated that the patients were in sinus rhythm.

The V^3^ trial^20^ and STRIVE-HF study^21^ recruited patients who were less likely to respond to conventional CRT. The V^3^ study only included non-responders to CRT, defined as unchanged or worsened clinical composite score at 6 months.^20^ The rationale was that multi-lead pacing may improve response in these patients, by increasing the chance of pacing the optimal site within the LV, or by increasing the volume of stimulated myocardium and reducing LV activation times. However, no clinical benefit for multi-lead pacing was observed over the control group who continued with a single LV lead. The location of the existing lead was not reported in the study, nor was the presence or location of scar. Thus it is difficult to ascertain if the lack of symptomatic response in this patient cohort was related to sub-optimal LV pacing. The STRIVE-HF study only included heart failure patients with LBBB and intermediate QRS prolongation (120–150 ms).^21^ The rationale for this was that these patients were less likely than those with LBBB and QRS >150 ms to respond to conventional CRT, and may benefit from the theoretically faster activation of the LV during multi-lead pacing.^2^ However, this study also did not demonstrate any benefit for multi-lead pacing over conventional CRT.

The pacing configuration also varied between groups. As previous discussed, while the majority of trials performed multi-lead pacing using two LV leads and one RV lead,^17^^,^^18^^,^^20^^,^^21^ one study investigated the effect of two RV leads in combination with one LV lead,^16^ and one study included both configurations in separate groups.^15^ It should be noted that in the latter study, by Rogers *et al*.,^15^ the configuration of two RV leads and one LV lead was only performed in patients where the implantation of two lead leads within the coronary sinus was not possible. These different configurations of multi-lead pacing are arguably very different in their effect on myocardial activation. As previously discussed, the implantation of two LV leads in the coronary sinus has the theoretical benefit of increasing the chance of stimulating the latest site of activation as well as stimulating a larger volume of myocardium. In the studies with two RV leads, one lead was positioned in the RV apex, while the second was positioned in a high septal position, and Anselme *et al*.^16^ specified this was ‘at or above the level of the His bundle’. It is therefore possible that the dual-site RV pacing performed in these studies involved at least non-selective stimulation of the conduction system, which is a very different physiological effect than that of dual-site pacing within the coronary sinus. While Anselme *et al*. did demonstrate some evidence for a beneficial effect of their multi-lead pacing configuration, with a higher proportion of echocardiographic responders in a subset of patients who had extended follow-up, there was no benefit observed in the study by Rodgers *et al*. for multi-lead pacing in Group B patient (two RV leads + one LV lead), with the overall study findings being driven by the benefit observed in Group A (two LV leads + one RV lead).^15^ Given the difference between the two configurations of multi-lead pacing, we performed sub-group analyses of all endpoints for patients with LV-only multi-lead pacing.

### Study limitations

The main limitations of the study are related to the variability of the studies included. While 415 patients were included in the study, the cohorts of patients in each trial differed. Therefore, it is difficult to generalize the findings of this meta-analysis to ‘all-comers’ who are indicated for CRT. Furthermore, while no overall benefit was found for multi-lead pacing when the studies were combined, it is possible that the additional of a third ventricular lead may be beneficial in specific subgroups of patients. For example, patients with permanent AF who require pacing for bradycardia did appear to benefit from multi-lead pacing in the TRIP-HF trial.^17^ In addition, while the STRIVE-HF study did not demonstrate a significant benefit for multi-lead pacing in a sub-analysis of patients with ischaemic cardiomyopathy, it may be under-powered to find a significant benefit in this sub-group.^21^ However, we were unable to report differential effects of multi-lead pacing in specific patient sub-groups within the meta-analysis due to lack of reporting by individual studies. Another limitation is the potential bias in the reporting of endpoint data. Only two studies reported blinding of the patients to the treatment group, and while most studies had blinded assessment of echocardiographic endpoints, only a minority reported blinding of assessors of symptomatic endpoints. There was also variability in how data were reported between studies. While two trials reported endpoints as improvement from baseline,^20^^,^^21^ the remainder reported absolute values at follow-up only. While the latter studies demonstrated no significant differences in baseline values, it is possible that variability between groups at baseline had an effect on the reported outcomes at follow-up.

## Conclusion

This meta-analysis does not demonstrate significant benefits for multi-lead pacing over conventional CRT. However, significant heterogeneity between studies in terms of inclusion criteria, trial design and multi-lead pacing configuration were noted, and it remains possible that multi-lead pacing is beneficial in selective patients. Further investigation may be warranted in sub-groups of patients undergoing CRT, such as those with atrial fibrillation or ischaemic cardiomyopathy.

## Data availability

The data underlying this article are available in the article and in its online supplementary material.

## Lead author biography

**Figure:**
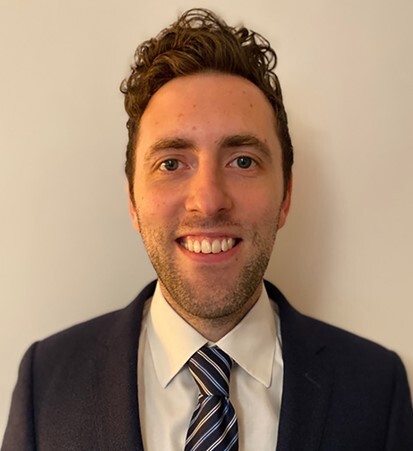


Mark K. Elliott is a cardiology trainee in the North East Thames deanery and a PhD research fellow working in the school of Biomedical Engineering and Imaging Sciences at King’s College London. His research interests include cardiac resynchronization therapy, endocardial left ventricular pacing, conduction system pacing, and electrocardiographic imaging.

## Funding

The department is supported by the Wellcome/EP SRC Centre for Medical Engineering (WT203148/Z/16/Z).

**Conflict of interest:** The department is supported by the Wellcome/EPSRC Centre for Medical Engineering (WT203148/Z/16/Z). Outside of the submitted work, B.S.S. is supported by a project grant from NIHR and has received speaker fees from EBR systems. M.K.E., V.M., and J.G. have received fellowship funding from Abbott. S.N. acknowledges support from the UK Engineering and Physical Sciences Research Council (EP/M012492/1, NS/A000049/1, and EP/P01268X/1), the British Heart Foundation (PG/15/91/31812, PG/13/37/30280, and SP/18/6/33805), US National Institutes of Health (NIH R01-HL152256), European Research Council (ERC PREDICT-HF 864055), and Kings Health Partners London National Institute for Health Research (NIHR) Biomedical Research Centre. C.A.R. receives research funding and/or consultation fees from Abbott, Medtronic, Boston Scientific, Spectranetics and MicroPort outside of the submitted work.

## References

[1] Sieniewicz BJ , Gould J, Porter B, Sidhu BS, Teall T, Webb J, Carr-White G, Rinaldi CA. Understanding non-response to cardiac resynchronisation therapy: common problems and potential solutions. Heart Fail Rev 2019;24:41–54. 30143910 10.1007/s10741-018-9734-8 PMC6313376

[2] Rinaldi CA , Burri H, Thibault B, Curnis A, Rao A, Gras D, Sperzel J, Singh JP, Biffi M, Bordachar P, Leclercq C. A review of multisite pacing to achieve cardiac resynchronization therapy. Europace 2015;17:7–17. 25214507 10.1093/europace/euu197

[3] Thibault B , Dubuc M, Khairy P, Guerra PG, Macle L, Rivard L, Roy D, Talajic M, Karst E, Ryu K, Paiement P, Farazi TG. Acute haemodynamic comparison of multisite and biventricular pacing with a quadripolar left ventricular lead. Europace 2013;15:984–991. 23447571 10.1093/europace/eus435

[4] Rinaldi CA , Leclercq C, Kranig W, Kacet S, Betts T, Bordachar P, Gutleben K-J, Shetty A, Donal E, Keel A, Ryu K, Farazi TG, Simon M, Naqvi TZ. Improvement in acute contractility and hemodynamics with multipoint pacing via a left ventricular quadripolar pacing lead. J Interv Card Electrophysiol 2014;40:75–80. 24626999 10.1007/s10840-014-9891-1

[5] Pappone C , Ćalović Ž, Vicedomini G, Cuko A, McSpadden LC, Ryu K, Romano E, Saviano M, Baldi M, Pappone A, Ciaccio C, Giannelli L, Ionescu B, Petretta A, Vitale R, Fundaliotis A, Tavazzi L, Santinelli V. Multipoint left ventricular pacing improves acute hemodynamic response assessed with pressure-volume loops in cardiac resynchronization therapy patients. Heart Rhythm 2014;11:394–401. 24291411 10.1016/j.hrthm.2013.11.023

[6] Mehta VS , Elliott MK, Sidhu BS, Gould J, Porter B, Niederer S, Rinaldi CA. Multipoint pacing for cardiac resynchronisation therapy in patients with heart failure: a systematic review and meta-analysis. J Cardiovasc Electrophysiol 2021;32:2577–2589. 34379350 10.1111/jce.15199 PMC7617165

[7] Antoniadis AP , Sieniewicz B, Gould J, Porter B, Webb J, Claridge S, Behar JM, Rinaldi CA. Updates in cardiac resynchronization therapy for chronic heart failure: review of multisite pacing. Curr Heart Fail Rep 2017;14:376–383. 28779280 10.1007/s11897-017-0350-z

[8] Yamasaki H , Seo Y, Tada H, Sekiguchi Y, Arimoto T, Igarashi M, Kuroki K, Machino T, Yoshida K, Murakoshi N, Ishizu T, Aonuma K. Clinical and procedural characteristics of acute hemodynamic responders undergoing triple-site ventricular pacing for advanced heart failure. Am J Cardiol 2011;108:1297–1304. 21855835 10.1016/j.amjcard.2011.06.048

[9] Yoshida K , Seo Y, Yamasaki H, Tanoue K, Murakoshi N, Ishizu T, Sekiguchi Y, Kawano S, Otsuka S, Watanabe S, Yamaguchi I, Aonuma K. Effect of triangle ventricular pacing on haemodynamics and dyssynchrony in patients with advanced heart failure: a comparison study with conventional bi-ventricular pacing therapy. Eur Heart J 2007;28:2610–2619. 17947217 10.1093/eurheartj/ehm441

[10] Ginks MR , Duckett SG, Kapetanakis S, Bostock J, Hamid S, Shetty A, Ma Y, Rhode KS, Carr-White GS, Razavi RS, Rinaldi CA. Multi-site left ventricular pacing as a potential treatment for patients with postero-lateral scar: insights from cardiac magnetic resonance imaging and invasive haemodynamic assessment. Europace 2012;14:373–379. 22045930 10.1093/europace/eur336

[11] Pappone C , Rosanio S, Oreto G, Tocchi M, Gulletta S, Salvati A, Dicandia C, Santinelli V, Mazzone P, Veglia F, Ding J, Sallusti L, Spinelli J, Vicedomini G. Cardiac pacing in heart failure patients with left bundle branch block: impact of pacing site for optimizing left ventricular resynchronization. Ital Heart J 2000;1:464–469. 10933328

[12] Sohal M , Shetty A, Niederer S, Lee A, Chen Z, Jackson T, Behar JM, Claridge S, Bostock J, Hyde E, Razavi R, Prinzen F, Rinaldi CA. Mechanistic insights into the benefits of multisite pacing in cardiac resynchronization therapy: the importance of electrical substrate and rate of left ventricular activation. Heart Rhythm 2015;12:2449–2457. 26165943 10.1016/j.hrthm.2015.07.012

[13] Lenarczyk R , Kowalski O, Kukulski T, Szulik M, Pruszkowska-Skrzep P, Zielinska T, Kowalczyk J, Pluta S, Duszanska A, Sredniawa B, Musialik-Lydka A, Kalarus Z. Triple-site biventricular pacing in patients undergoing cardiac resynchronization therapy: a feasibility study. Europace 2007;9:762–767. 17631515 10.1093/europace/eum140

[14] Lenarczyk R , Kowalski O, Kukulski T, Pruszkowska-Skrzep P, Sokal A, Szulik M, Zielińska T, Kowalczyk J, Pluta S, Średniawa B, Musialik-Łydka A, Kalarus Z. Mid-term outcomes of triple-site vs. conventional cardiac resynchronization therapy: a preliminary study. Int J Cardiol 2009;133:87–94. 18242737 10.1016/j.ijcard.2007.12.009

[15] Rogers DPS , Lambiase PD, Lowe MD, Chow AWC. A randomized double-blind crossover trial of triventricular versus biventricular pacing in heart failure. Eur J Heart Fail 2012;14:495–505. 22312038 10.1093/eurjhf/hfs004

[16] Anselme F , Bordachar P, Pasquié JL, Klug D, Leclercq C, Milhem A, Alonso C, Deharo JC, Gras D, Probst V, Piot O, Savouré A. Safety, feasibility, and outcome results of cardiac resynchronization with triple-site ventricular stimulation compared to conventional cardiac resynchronization. Heart Rhythm 2016;13:183–189. 26325531 10.1016/j.hrthm.2015.08.036

[17] Leclercq C , Gadler F, Kranig W, Ellery S, Gras D, Lazarus A, Clémenty J, Boulogne E, Daubert J-C; TRIP-HF (Triple Resynchronization In Paced Heart Failure Patients) Study Group. A randomized comparison of triple-site versus dual-site ventricular stimulation in patients with congestive heart failure. J Am Coll Cardiol 2008;51:1455–1462. 18402900 10.1016/j.jacc.2007.11.074

[18] Lenarczyk R , Kowalski O, Sredniawa B, Pruszkowska-Skrzep P, Mazurek M, Jędrzejczyk-Patej EWA, Woźniak A, Pluta S, Głowacki JAN, Kalarus Z. Implantation feasibility, procedure-related adverse events and lead performance during 1-year follow-up in patients undergoing triple-site cardiac resynchronization therapy: a substudy of TRUST CRT randomized trial. J Cardiovasc Electrophysiol 2012;23:1228–1236. 22651239 10.1111/j.1540-8167.2012.02375.x

[19] Zhang B , Guo J, Zhang G. Comparison of triple-site ventricular pacing versus conventional cardiac resynchronization therapy in patients with systolic heart failure: a meta-analysis of randomized and observational studies. J Arrhythm 2018;34:55–64. 29721114 10.1002/joa3.12018 PMC5828262

[20] Bordachar P , Gras D, Clementy N, Defaye P, Mondoly P, Boveda S, Anselme F, Klug D, Piot O, Sadoul N, Babuty D, Leclercq C. Clinical impact of an additional left ventricular lead in cardiac resynchronization therapy nonresponders: the V3 trial. Heart Rhythm 2018;15:870–876. 29288035 10.1016/j.hrthm.2017.12.028

[21] Gould J , Claridge S, Jackson T, Sieniewicz BJ, Sidhu BS, Porter B, Elliott MK, Mehta V, Niederer S, Chadwick H, Kamdar R, Adhya S, Patel N, Hamid S, Rogers D, Nicolson W, Chan CF, Whinnett Z, Murgatroyd F, Lambiase PD, Rinaldi CA. Standard care vs. TRIVEntricular pacing in Heart Failure (STRIVE HF): a prospective multicentre randomized controlled trial of triventricular pacing vs. conventional biventricular pacing in patients with heart failure and intermediate QRS left bundle branch block. Europace 2021;1–11. 10.1093/europace/euab267. 35079787 PMC9071069

[22] Moher D , Liberati A, Tetzlaff J, Altman DG; for the PRISMA Group. Preferred reporting items for systematic reviews and meta-analyses: the PRISMA statement. BMJ (Online) 2009;339:b2535. 10.1136/bmj.b2535 PMC2714657 19622551

[23] Sterne JAC , Savović J, Page MJ, Elbers RG, Blencowe NS, Boutron I, Cates CJ, Cheng H-Y, Corbett MS, Eldridge SM, Emberson JR, Hernán MA, Hopewell S, Hróbjartsson A, Junqueira DR, Jüni P, Kirkham JJ, Lasserson T, Li T, McAleenan A, Reeves BC, Shepperd S, Shrier I, Stewart LA, Tilling K, White IR, Whiting PF, Higgins JPT. RoB 2: a revised tool for assessing risk of bias in randomised trials. BMJ 2019;366:l4898. 31462531 10.1136/bmj.l4898

[24] Deeks JJ , Higgins JPT, Altman DG. Analysing data and undertaking meta-analyses. In: Higgins JPT, Thomas J, Chandler J, et al. eds. *Cochrane Handbook for Systematic Reviews of Interventions*, Vol. 2. Cochrane John Wiley & Sons Ltd., 2019, pp. 241–284. 10.1002/9781119536604.ch10.

[25] Higgins JPT , Thompson SG, Deeks JJ, Altman DG. Measuring inconsistency in meta-analyses. Br Med J 2003;327:557–560. 12958120 10.1136/bmj.327.7414.557 PMC192859

[26] Lenarczyk R , Kowalski O, Sredniawa B, Pruszkowska-Skrzep P, Pluta S, Sokal ADAM, Kukulski T, Stabryła-Deska J, Woźniak A, Kowalczyk J, Zielińska T, Mazurek M, Streb W, Zembala M, Kalarus Z. Triple-site versus standard cardiac resynchronization therapy study (TRUST CRT): clinical rationale, design, and implementation. J Cardiovasc Electrophysiol 2009;20:658–662. 19635069 10.1111/j.1540-8167.2008.01394.x

[27] Akerström F , Narváez I, Puchol A, Pachón M, Martín-Sierra C, Rodríguez-Mañero M, Rodríguez-Padial L, Arias MA. Estimation of the effects of multipoint pacing on battery longevity in routine clinical practice. Europace 2018;20:1161–1167. 29036370 10.1093/europace/eux209

[28] D'Onofrio A , Bertini M, Infusino T, D'Arienzo G, Cipolletta L, Bianchi V, Licciardello G, Savarese G, Russo G, Ricciardi D, Manzo M, Fabbri F, Notarstefano P, Santini L, Campari M, Valsecchi S, Forleo GB. Single- and multi-site pacing strategies for optimal cardiac resynchronization therapy: impact on device longevity and therapy cost. J Interv Card Electrophysiol 2021;60:195–203. 32185588 10.1007/s10840-020-00711-3

[29] Rho RW , Patel VV, Gerstenfeld EP, Dixit S, Poku JW, Ross HM, Callans D, Kocovic DZ. Elevations in ventricular pacing threshold with the use of the Y adaptor: implications for biventricular pacing. Pacing Clin Electrophysiol 2003;26:747–751. 12698677 10.1046/j.1460-9592.2003.00127.x

[30] Mayhew MW , Johnson PL, Slabaugh JE, Bubien RS, Kay GN. Electrical characteristics of a split cathodal pacing configuration. Pacing Clin Electrophysiol 2003;26:2264–2271. 14675010 10.1111/j.1540-8159.2003.00357.x

[31] Ploux S , Strik M, van Hunnik A, van Middendorp L, Kuiper M, Prinzen FW. Acute electrical and hemodynamic effects of multisite left ventricular pacing for cardiac resynchronization therapy in the dyssynchronous canine heart. Heart Rhythm 2014;11:119–125. 24120876 10.1016/j.hrthm.2013.10.018

[32] Heckman LIB , Kuiper M, Anselme F, Ziglio F, Shan N, Jung M, Zeemering S, Vernooy K, Prinzen FW. Evaluating multisite pacing strategies in cardiac resynchronization therapy in the preclinical setting. Heart Rhythm O2 2020;1:111–119. 34113865 10.1016/j.hroo.2020.03.003 PMC8183878

[33] Jackson T , Lenarczyk R, Sterlinski M, Sokal A, Francis D, Whinnett Z, Van Heuverswyn F, Vanderheyden M, Heynens J, Stegemann B, Cornelussen R, Rinaldi CA. Left ventricular scar and the acute hemodynamic effects of multivein and multipolar pacing in cardiac resynchronization. Int J Cardiol Heart Vasc 2018;19:14–19. 29946558 10.1016/j.ijcha.2018.03.006 PMC6016076

[34] Wilton SB , Leung AA, Ghali WA, Faris P, Exner D. V. Outcomes of cardiac resynchronization therapy in patients with versus those without atrial fibrillation: a systematic review and meta-analysis. Heart Rhythm 2011;8:1088–1094. 21338711 10.1016/j.hrthm.2011.02.014

[35] Gervais R , Leclercq C, Shankar A, Jacobs S, Eiskjaer H, Johannessen A, Freemantle N, Cleland JGF, Tavazzi L, Daubert C; on behalf of the CARE-HF investigators. Surface electrocardiogram to predict outcome in candidates for cardiac resynchronization therapy: a sub-analysis of the CARE-HF trial. Eur J Heart Fail 2009;11:699–705. 19505883 10.1093/eurjhf/hfp074

[36] Lin J , Buhr KA, Kipp R. Effect of PR interval on outcomes following cardiac resynchronization therapy: a secondary analysis of the COMPANION trial. J Cardiovasc Electrophysiol 2017;28:185–191. 27885751 10.1111/jce.13131

[37] Kutyifa V , Stockburger M, Daubert JP, Holmqvist F, Olshansky B, Schuger C, Klein H, Goldenberg I, Brenyo A, McNitt S, Merkely B, Zareba W, Moss AJ. PR interval identifies clinical response in patients with non-left bundle branch block a multicenter automatic defibrillator implantation trial-cardiac resynchronization therapy substudy. Circ Arrhythm Electrophysiol 2014;7:645–651. 24963007 10.1161/CIRCEP.113.001299

[38] Glikson M , Nielsen JC, Kronborg MB, Michowitz Y, Auricchio A, Barbash IM, Barrabés JA, Boriani G, Braunschweig F, Brignole M, Burri H, Coats AJS, Deharo JC, Delgado V, Diller GP, Israel CW, Keren A, Knops RE, Kotecha D, Leclercq C, Merkely B, Starck C, Thylén I, Tolosana JM; ESC Scientific Document Group. 2021 ESC Guidelines on cardiac pacing and cardiac resynchronization therapy: developed by the Task Force on cardiac pacing and cardiac resynchronization therapy of the European Society of Cardiology (ESC) With the special contribution of the European Hear. Eur Heart J 2021. 10.1093/eurheartj/ehab364.

[39] Yancy CW , Jessup M, Bozkurt B, Butler J, Casey DE, Drazner MH, Fonarow GC, Geraci SA, Horwich T, Januzzi JL, Johnson MR, Kasper EK, Levy WC, Masoudi FA, McBride PE, McMurray JJV, Mitchell JE, Peterson PN, Riegel B, Sam F, Stevenson LW, Tang WHW, Tsai EJ, Wilkoff BL. 2013 ACCF/AHA guideline for the management of heart failure: a report of the American College of Cardiology Foundation/American Heart Association Task Force on Practice Guidelines. Circulation 2013;128:240–327. 10.1161/CIR.0b013e31829e8776 23741058

